# The relationship between sleep duration and thyroid function in the adult US population: NHANES 2007–2012

**DOI:** 10.1371/journal.pone.0291799

**Published:** 2023-09-21

**Authors:** Mingzheng Wang, Xiaofeng Lu, Xiaogang Zheng, Chaoyang Xu, Junru Liu

**Affiliations:** 1 Department of Breast and Thyroid, Jinhua Central Hospital, Jinhua, Zhejiang, China; 2 Department of Endocrinology and Metabolism, Jinhua People’s Hospital, Jinhua, Zhejiang, China; King Abdulaziz University Faculty of Medicine, SAUDI ARABIA

## Abstract

**Objective:**

Sleep disturbance is a common problem in the general population. Sleep deprivation or dysfunction can have profound health consequences. However, how sleep duration is associated with thyroid function remains unclear. This study was thus developed to examine the association between sleep duration and thyroid function in the US adult population.

**Methods:**

A total of 8102 participants from the NHANES 2007–2012 dataset were included in this study. Weighted data analyses were conducted, and the link between sleep duration and thyroid function was probed using linear regression models with smoothed curve fitting. Stratified analyses were also performed.

**Results:**

Weighted mean (standard deviation) values for study variables were as follows: sleep duration 6.85 (0.02) hours, thyroid-stimulating hormone (TSH) 1.86 (0.03) mIU/ml, serum free T3 3.20 (0. 01) pg/mL, serum free T4 0.80 (0.01) ng/dL, serum total T3 115.12 (0.64) ng/dL, serum total T4 7.81 (0.04) ug/dL, TPOAb 16.20 (1.53) IU/mL, TgAb 5.75 (0.73) IU/mL, and Tg 15.11 (0.46) ng/mL. In unadjusted analyses, increased sleep duration was associated with higher serum TSH levels and decreased FT3 levels. After adjustment for potential confounders, a significant negative relationship was detected between sleep duration and FT3 levels in participants with ≤7 hours of sleep. When sleep duration exceeded 7 hours, no significant changes in FT3 levels were observed after further increases in sleep duration.

**Conclusion:**

Increased sleep duration was related to decreased FT3 levels, primarily at short sleep durations, and this correlation was no longer evident when participants reached the recommended healthy sleep duration.

## Introduction

Sleep is essential for human health, affecting a wide range of key physiological functions [1, 2]. Sleep is complex and subject to tight physiological regulation [3]. The recommend daily duration of sleep for adults is 7–9 hours [4, 5]. However, millions of Americans suffer from sleep-related disorders each year, with only 48% of adults reporting a habitual sleep duration within this range [6]. Similar patterns of gradually decreasing sleep duration have been observed in several Western nations over the past few decades [7]. Many studies have demonstrated that insufficient or disturbed sleep has far-reaching implications for health. A close relationship between sleep duration and health conditions such as cardiovascular events [8–10], mental disorders [11, 12], and mortality [13] has been observed in several epidemiological studies.

Thyroid hormone (TH) production and signaling activity plays a key role in human growth and development, shaping physiological processes such as digestion, respiration, heart rate, and thermoregulation [14]. The hypothalamic-pituitary-thyroid (HPT) axis tightly controls TH production and secretion. This central axis initiates TH production based on signaling in the hypothalamic paraventricular nucleus prior to signal transmission through the pituitary and thyroid glands [15, 16]. In addition, almost all hormones are produced in a cyclical rhythm over 24-hour intervals, with sleep having a varying impact on the regulation of this rhythm [17–19]. Notably, sleep strongly impacts thyroid-stimulating hormone (TSH) with respect to both the quality and duration of sleep, and free triiodothyronine (FT3) also showed a 24-hour circadian rhythm parallel to TSH, but delayed [20, 21]. How sleep impacts the HPT axis has been suggested to be dependent on the period of sleep restriction [22], with short-term sleep restriction increasing secretion of TSH [23] and FT4 [24], but long-term sleep restriction suppressing TSH and FT4 secretion [25], but the sample size is relatively small.

Currently, the relationship between sleep duration and thyroid function profiles remains unclear, and few large-sample epidemiological analyses have examined the association between sleep duration and thyroid function in the general adult population. Therefore, the aim of this study was to clarify how weekday sleep duration relates to thyroid function in the US adult population.

## Methods

### Study population

The cross-sectional National Health and Nutrition Examination Survey (NHANES) compiles large volumes of data pertaining to the demographic characteristics, healthy behaviors, and nutrition of individuals in the US. Data from the NHANES are accessible for researcher use online, along with corresponding statistics (www.cdc.gov/nchs/nhanes/). The present study was performed as per the Declaration of Helsinki.

**Fig 1:**
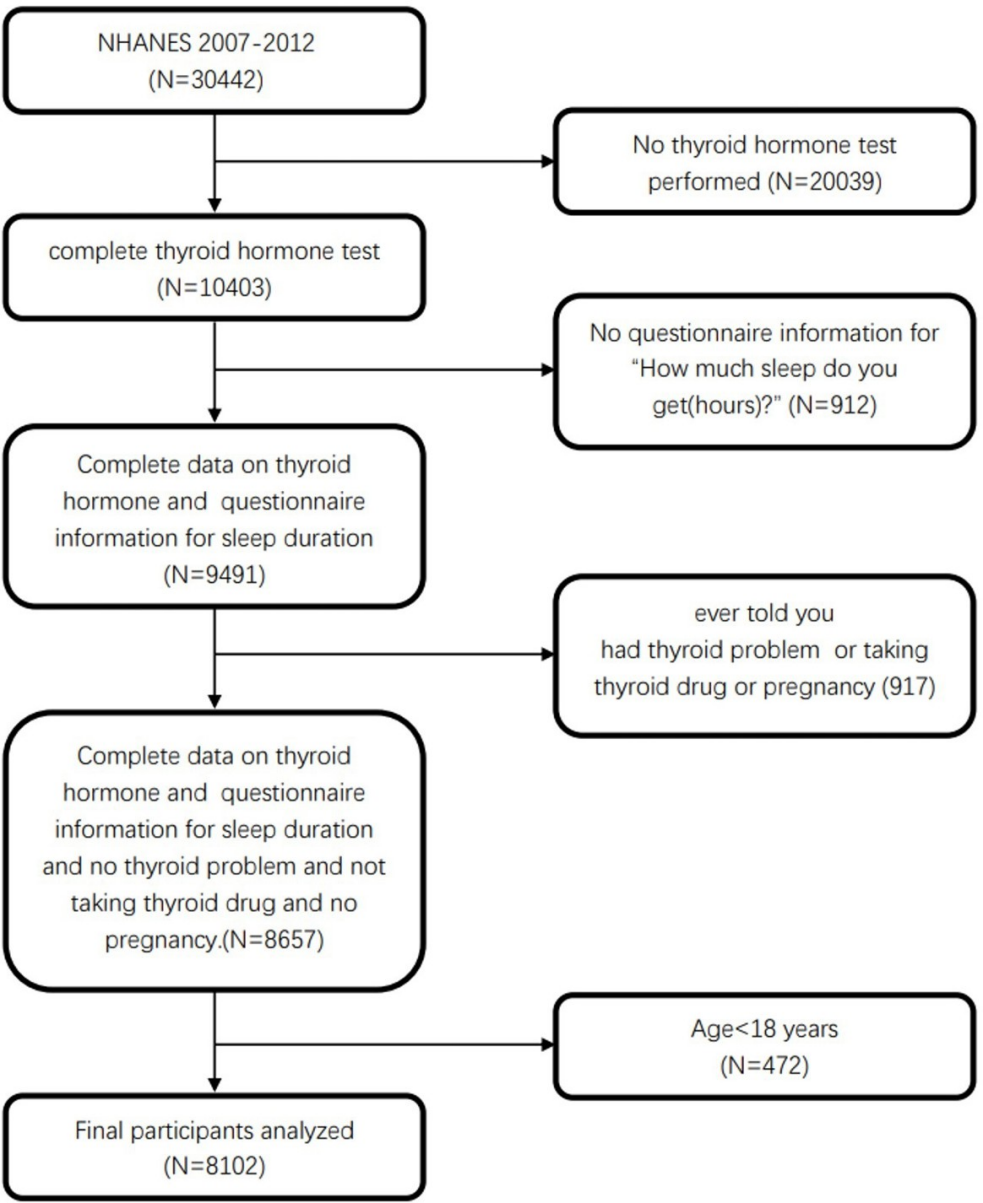
Study flowchart. NHANES, National Health and Nutrition Examination Survey.

The present study utilized NHANES 2007–2012 data, which received approval from the NCHS Research Ethics Review Board (ERB). All surveyed NHANES participants had provided written informed consent. For these analyses, 8,102 subjects ≥ 18 years of age for whom complete sleep- and thyroid function-related data were available were selected for inclusion. For further details regarding participant screening, see Fig 1.

### Measurements

#### Sleep duration

The assessment of sleep duration was based on the ’sleep disturbance’ data from the NHANES questionnaire. Question SLQ010H asked “How much sleep do you usually get at night on weekdays or workdays?”, with participants being requested to respond with a number of hours. Participants responded with their sleep duration from 2–11 hours. If they reported 12 or more hours, then a duration of 12 hours was recorded. Sleep duration was analyzed as a continuous and categorical variable. Based on the sleep duration recommendation of the National Sleep Foundation in the US and the consensus recommendations of the American Academy of Sleep Medicine and the Sleep Research Society regarding the duration of sleep necessary to promote optimal health in adults, sleep duration was stratified into three groups: < 7 h, 7–9 h, and >9 h [4, 26].

#### Thyroid outcomes

The thyroid parameters analyzed, including TSH, FT3, FT4, TT3, TT4, Tg, TgAb, and TPOAb levels, were determined using the Access 2 method (Beckman Coulter, from which the immunization reagents were purchased). Thyroid parameters were measured from 2007–2012. Participants were asked to fast for 9 hours and have their blood drawn within 4 hours of the morning, have their blood collected and processed for freezing and storage, and then have the collected blood samples sent together to a designated laboratory once a week for testing. TSH levels were analyzed using a third-generation two-site sandwich immunoassay with a reference range of 0.34–5.6 IU/mL. The reference range for the competitive binding immunoassay used to measure FT3 levels was 2.5–3.9 pg/mL, while the reference range for the two-step enzyme immunoassay used to measure FT4 levels was 0.6–1.6 ng/dL. Levels of TT3 and TT4 were also measured via competitive binding immunoenzymatic assays with respective reference ranges of 5.0–12.0 ug/dL and 80–220 ng/dL, respectively. A sequential two-step sandwich immunoassay was also used to measure TPOAb and TgAb titers, with respective 0–9.0 IU/mL and 0–4.0 IU/mL reference ranges. The Access Tg assay is a one-step simultaneous sandwich assay. The NHANES Laboratory/Medical Technologist Procedures Manual provides comprehensive instructions for specimen collection and processing.

#### Covariates

Participant age, race/ethnicity, poverty income ratio (PIR), smoking history, gender, and history of alcohol consumption were assessed using a standard questionnaire. Weight and height measurements, which are necessary for calculating participants’ body mass index (BMI), were collected by trained technicians in a mobile examination center (MEC). Additionally, considering the importance of iodine for the synthesis of thyroxine, urine samples were collected to determine participants’ urinary iodine concentration (UIC). The collected urine undergoes processing, storage, and is centrally shipped once a week to a designated laboratory for UIC testing (Inductively Coupled Plasma Dynamic Reaction Cell Mass Spectrometry, ICP-DRC-MS). Ethnicity was categorized into four groups (non-Hispanic black, non-Hispanic white, Mexican American, or other). Socioeconomic status was assessed using PIR values. BMI values were calculated by dividing participant weight (kg) by height squared (m^2^). Marital status was defined as a categorical variable with three groups: married or living with a partner; widowed, divorced, or separated; or never married. Alcohol consumption history was also categorized as never (< 12 lifetime drinks), former (≥ 12 drinks in 1 year but no drinks in the last year or ≥ 12 lifetime drinks but no drinks in the last year), light (≤ 2 and ≤ 1 daily drinks in males and females, respectively, in the last 12 months), moderate (3 or 2 daily drinks in males and females, respectively, in the last 12 months) or heavy (≥ 4 or ≥ 3 daily drinks in males and females, respectively, in the last 12 months). Smoking status was classified as never (< 100 lifetime cigarettes), former (> 100 lifetime cigarettes but not a current smoker), or current.

### Statistical analysis

The analytical guidelines (NHANES:2007–2012) were used when calculating and analyzing weighted data. The NHANES data release file includes multiple sample weights, including interview weight (wtint2yr), or examination weight (wtmec2yr), and the most appropriate weighting is dependent on the selected variables of interest. Given that the MEC sample is an interview sample subset, this analysis was conducted with the combined MEC examination weight. However, the thyroid examination sample from NHANES 2009–2012 (two survey cycles) is a subset of the overall participants that were interviewed for the MEC examination. As a result, the subset weight (WTSA2YR) was used when analyzing data from these two survey cycles. The weight of the survey allows it to be extended to the civilian noninstitutionalized US population [27, 28].

The association between sleep duration and thyroid function was described with both unadjusted and adjusted linear regression models. Model 1 did not adjust for covariates, model 2 adjusted for gender, age, race/ethnicity, BMI, alcohol consumption, smoking status, marital status and UIC levels of participants. Results were further resolved with smooth curve fitting. Stratified analyses were also performed to interrogate the independent effect of sleep duration on thyroid function levels. Data are presented as means with standard deviations (SDs) or percentages, as appropriate. Effect values are reported as β values with corresponding 95% confidence intervals (CIs). P < 0.05 served as the cut-off for statistical significance. All analyses were performed with R v4.2.2 with the ’nhanesR’ package (v0.9.4.1) and the ’survey’ package (v 4.1–1).

## Results

### Baseline participant characteristics

Table 1 describes the characteristics of participants grouped according to sleep duration. Of these 8102 participants, 4649 (weighted percentage: 57.38%) slept 7–9 h, 3241 (weighted percentage: 40%) slept < 7 h, and 212 (weighted percentage: 2.62%) slept > 9 h. The weighted mean (SD) sleep duration of these participants was 6.85 (0.02) h. The mean TSH levels of these participants was 1.86 (0.03) mIU/mL, with serum free T3 levels of 3.20 (0.01) pg/mL, serum free T4 levels of 0.80 (0.01) ng/dL, serum total T3 levels of 115. 12 (0.64) ng/dL, serum total T4 levels of 7.81 (0.04) ug/dL, TPOAb levels of 16.20 (1.53) IU/mL, TgAb levels of 5.75 (0.73) IU/mL, and Tg levels of 15.11 (0.46) ng/mL. ANOVAs revealed significant differences in TSH and FT3 levels among sleep duration subgroups, with higher TSH and lower FT3 levels in the longer sleep duration group. The study did not include participants who were reported to have thyroid disease, who were taking thyroid medication, or who were pregnant. Our study did not exclude participants who were not clinically diagnosed with abnormal thyroid function.

**Table 1:** Thyroid function of NHANES (2007–2012) study population in sleep duration groups.

Characteristics	Sleep duration				*P*-value
	total	<7 h	7–9 h	>9 h	
*N*	8102	3241	4649	212	
Sleep duration(h)	6.85(0.02)^a^	5.48(0.02)	7.60(0.02)	10.49(0.09)	< 0.001
Sex					0.13
Male	4318(53.3)^b^	1761(54.78)	2454(51.74)	103(45.45)	
Female	3784(46.7)	1480(45.22)	2195(48.26)	109(54.55)	
Race/Ethnicity					< 0.001
White	3554(43.87)	1269(62.21)	2189(69.97)	96(60.05)	
Black	1673(20.65)	889(15.93)	750 (8.25)	34(10.30)	
Mexican	1348(16.64)	460 (7.52)	856 (9.21)	32(10.57)	
Other	1527(18.85)	623(14.34)	854(12.57)	50(19.08)	
Age (years)	44.99(0.45)	44.43(0.53)	45.33(0.48)	45.43(3.37)	0.26
Ever told doctor had trouble sleeping?					< 0.001
No	6333(78.2)	2224(66.49)	3930(81.78)	179(88.30)	
Yes	1765(21.8)	1014(33.51)	718(18.22)	33(11.70)	
Ever told by doctor have sleep disorder?					< 0.001
No	7514(92.93)	2914(89.51)	4406(94.32)	194(92.94)	
Yes	572(7.07)	319(10.49)	236 (5.68)	17 (7.06)	
BMI (kg/m2)	28.37(0.14)	29.04(0.18)	27.99(0.18)	27.32(0.93)	< 0.001
TSH (mIU/L)	1.86(0.03)	1.79(0.04)	1.90(0.04)	2.11(0.14)	0.02
FT3 (pg/mL)	3.20(0.01)	3.24(0.02)	3.18(0.01)	3.14(0.04)	0.002
FT4 (ng/dL)	0.80(0.00)	0.80(0.01)	0.80(0.01)	0.82(0.01)	0.12
TT3 (ng/dL)	115.12(0.64)	116.11(0.85)	114.51(0.70)	114.57(2.81)	0.18
TT4 (ug/dL)	7.81(0.04)	7.81(0.05)	7.80(0.04)	8.09(0.12)	0.06
TPOAb (IU/mL)	16.20(1.53)	17.19(2.94)	15.62(1.57)	14.73(5.33)	0.84
TgAb (IU/mL)	5.75(0.73)	5.46(1.26)	5.88(0.82)	7.55(4.35)	0.9
Tg (ng/mL)	15.11(0.46)	15.68(0.70)	14.69(0.66)	16.73(1.46)	0.28
UIC (ug/dL)	236.41(11.42)	255.00(19.53)	225.50(12.64)	206.03(21.32)	0.13
Smoke					< 0.001
Never	4085(53.21)	1596(51.17)	2399(56.44)	90(52.48)	
Former	1881(24.5)	723(21.83)	1116(25.33)	42(19.61)	
Now	1711(22.29)	806(27.00)	850(18.22)	55(27.91)	
Alcohol.User					< 0.001
Never	982(13.79)	383 (9.54)	555 (9.96)	44(22.66)	
Former	1353(19.01)	608(16.84)	698(13.91)	47(24.31)	
Mild	2182(30.65)	838(31.84)	1308(34.76)	36(20.90)	
Moderate	1041(14.62)	414(15.73)	616(18.49)	11 (8.06)	
Heavy	1561(21.93)	635(26.05)	890(22.87)	36(24.06)	
Poverty-to-income ratio	2.93(0.05)	2.76(0.08)	3.06(0.06)	2.26(0.15)	< 0.001
Marital					< 0.001
Never married	1391(18.11)	575(20.63)	779(18.39)	37(21.05)	
Widowed, divorced, or separated	1668(21.72)	772(19.45)	836(15.35)	60(22.81)	
Married, or living with partner	4622(60.17)	1778(59.91)	2754(66.26)	90(56.14)	

^a^ Continuous Variables: weighted mean (SD).

^b^ Categorical variable: actual frequency (weighted percentage).

Abbreviations: FT3, free triiodothyronine; FT4, free thyroxine; TSH, thyroid-stimulating hormone; TT3, total T3; TT4, total T4; Tg, thyroglobulin; TPOAb, thyroid peroxidase antibody; BMI, body mass index; UIC, urinary iodine concentration; SD, Standard deviation.

### The association between thyroid function and sleep duration

Table 2 shows that in an unadjusted model, TSH was positively associated with sleep duration (β = 0.056, 95% CI: 0.023 to 0.089, P = 0.001). When sleep duration was categorized into 3 groups (<7h, 7-9h, and >9h), this positive association was still present (P for trend = 0.005), but this association did not remain after adjusting for confounders in model 2. In contrast, FT3 levels were negatively associated with sleep duration. This negative association remained after further adjustment for confounders in model 2 (β = -0.017, 95% CI: -0.028 to -0.005, P = 0.005) and after dividing sleep duration into 3 groups (P for trend = 0.013).

**Table 2:** The association between sleep duration and thyroid function.

	Model 1^a^	*p*-value	Model 2^b^	*p*-value
	*β* (95% CI)		*β* (95% CI)	
**TSH(mIU/L)**				
Sleep duration(h)	0.056(0.023,0.089)	0.001	0.029(-0.011,0.068)	0.149
categories				
<7 h	Reference		Reference	
7–9 h	0.110(0.022,0.198)	0.016	-0.002(-0.108,0.103)	0.962
>9 h	0.319(0.022,0.616)	0.036	0.328(-0.068,0.725)	0.101
p for trend		0.005		0.624
**FT3(pg/mL)**				
Sleep duration(h)	-0.021(-0.032,-0.009)	<0.001	-0.017(-0.028,-0.006)	0.005
categories				
<7 h	Reference		Reference	
7–9 h	-0.060(-0.098,-0.023)	0.002	-0.046(-0.083,-0.009)	0.016
>9 h	-0.102(-0.176,-0.027)	0.009	-0.072(-0.141,-0.002)	0.044
p for trend		<0.001		0.013
**FT4(ng/dL)**				
Sleep duration(h)	0.002(-0.001,0.006)	0.169	0.001(-0.004,0.005)	0.745
categories				
<7 h	Reference		Reference	
7–9 h	0.003(-0.006,0.013)	0.447	-0.002(-0.012,0.009)	0.750
>9 h	0.027(0.001,0.054)	0.045	0.012(-0.021,0.045)	0.461
p for trend		0.192		0.935
**TT3(ng/dL)**				
Sleep duration(h)	-0.433(-1.052,0.185)	0.166	-0.244(-0.866,0.378)	0.430
categories				
<7 h	Reference		Reference	
7–9 h	-1.594(-3.373,0.184)	0.078	-0.684(-2.385,1.017)	0.418
>9 h	-1.540(-6.769,3.688)	0.556	-2.628(-6.544,1.289)	0.181
p for trend		0.065		0.320
**TT4(ug/dL)**				
Sleep duration(h)	0.015(-0.025,0.055)	0.449	0.020(-0.023,0.064)	0.352
categories				
<7 h	Reference		Reference	
7–9 h	-0.007(-0.117,0.102)	0.893	0.032(-0.093,0.158)	0.601
>9 h	0.280(0.049,0.510)	0.018	0.140(-0.109,0.389)	0.259
p for trend		0.658		0.472
**TPOAb(IU/mL)**				
Sleep duration(h)	0.360(-2.042,2.762)	0.764	-0.554(-3.771,2.664)	0.728
categories				
<7 h	Reference		Reference	
7–9 h	-1.565(-8.009,4.880)	0.628	-4.777(-12.091,2.536)	0.192
>9 h	-2.458(-13.876,8.961)	0.667	-1.471(-19.320,16.379)	0.867
p for trend		0.591		0.214
**TgAb (IU/mL)**				
Sleep duration(h)	0.371(-0.415,1.157)	0.348	-0.056(-1.057,0.946)	0.910
categories				
<7 h	Reference		Reference	
7–9 h	0.417(-2.459,3.294)	0.772	-0.512(-3.899,2.875)	0.759
>9 h	2.082(-7.351,11.514)	0.659	1.865(-12.566,16.296)	0.793
p for trend		0.705		0.875
**Tg (ng/mL)**				
Sleep duration(h)	-0.292(-1.271,0.687)	0.552	-0.020(-1.192,1.152)	0.973
categories				
<7 h	Reference		Reference	
7–9 h	-0.997(-3.006,1.012)	0.323	0.076(-1.992,2.144)	0.941
>9 h	1.044(-2.203,4.291)	0.521	0.514(-3.986,5.013)	0.817
p for trend		0.435		0.908

^**a**^ Model 1: no covariates were adjusted.

^**b**^ Model 2: age, gender, race/ethnicity, poverty-to-income ratio, marital, body mass index, alcohol use, smoke, urinary iodine concentration, sleep disorder(ever told doctor had trouble sleeping or ever told by doctor have sleep disorder) were adjusted.

Abbreviations: TSH, thyroid-stimulating hormone; FT3, free triiodothyronine; FT4, free thyroxine; TT3, total T3; TT4, total T4; TPOAb, anti-thyroid peroxidase antibody; TgAb, anti-thyroglobulin antibody; Tg, thyroglobulin.

**Fig 2:**
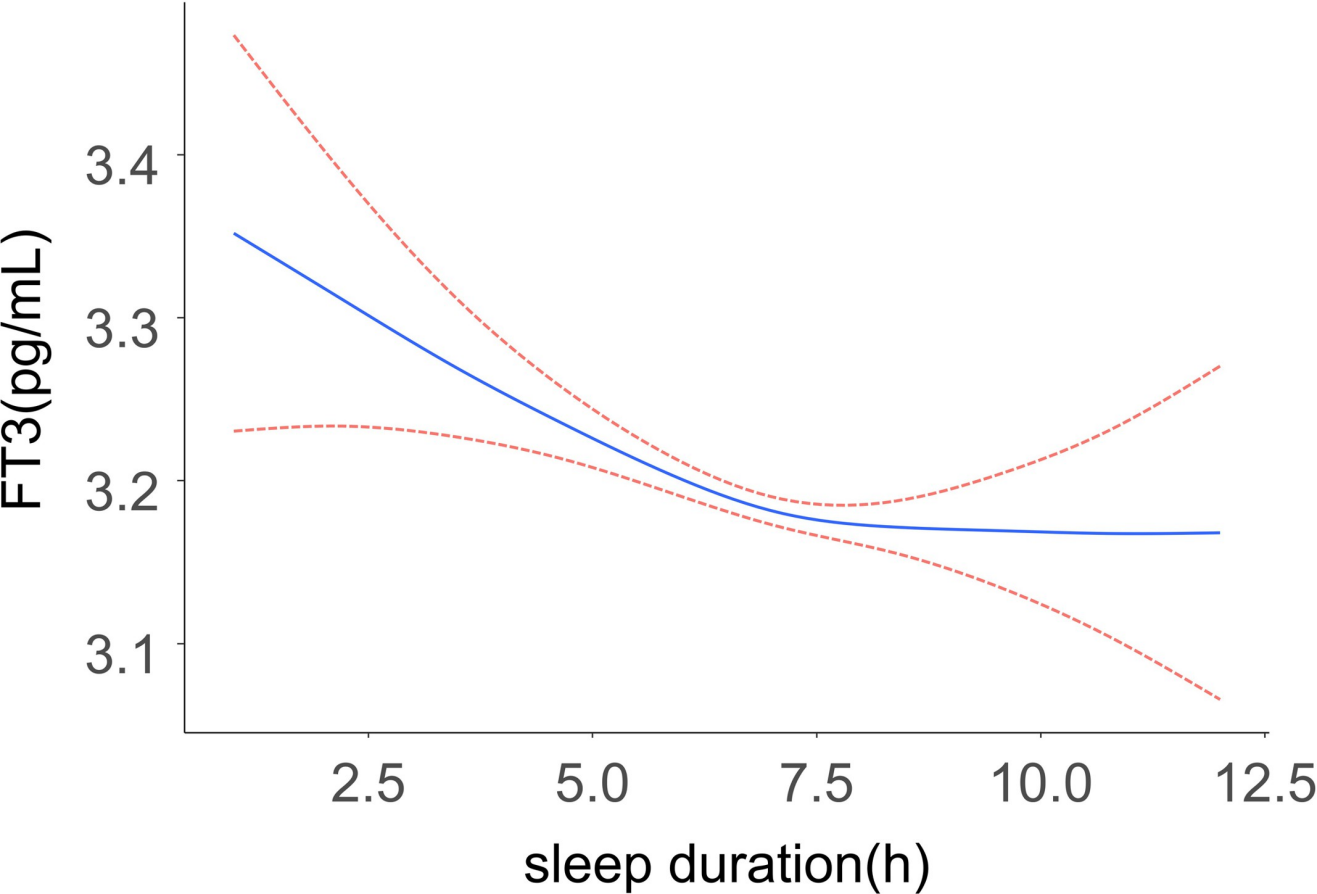
A flat L-shaped association between sleep duration and FT3 was found in the generalized additive model (GAM). The solid line represents the smoothed curve fit between the variables and the dashed line represents the 95% confidence interval of the fit. The model was adjusted for sex, age, race/ethnicity, marital status, BMI, sleep disturbance, poverty income ratio, smoking status, alcohol consumption status, UIC.

As shown in Fig 2, a generalized additive model revealed a flat L-shaped relationship between sleep duration and FT3 levels. FT3 levels were found to decrease with increasing sleep duration in participants with short sleep duration, but as sleep duration continued to increase after reaching a healthy sleep duration, FT3 levels leveled off and no longer decreased significantly. The solid line represents the smoothed curve fit between the variables while 95% CI for this fit is represented with a dashed line. Results were adjusted for all covariates (sex, age, race/ethnicity, marital status, BMI, sleep disturbance, poverty income ratio, smoking status, alcohol consumption status, UIC). A log-likelihood ratio test was also conducted, and comparisons of two-segment and one-line regression models were performed. A two-step recursive method revealed an inflection point of K = 7 h. For further details regarding these results, see Table 3.

**Table 3:** Analysis of sleep duration and FT3 using segmented linear regression.

Models	β (95%CI)	*P* value
**Model I**		
One line effect	-0.017(-0.028,-0.006)	0.005
**Model II**		
Inflection point (K)	7 h	
sleep duration ≤ 7 h	-0.032 (-0.053, -0.011)	0.004
sleep duration > 7 h	0.006 (-0.022, 0.033)	0.664
P for log-likelihood ratio test		0.026

Model I, one-line linear regression; Model II, two-segment regression

β was the effect size and the 95% CI indicated the confidence interval

adjust for: sex, age, race/ethnicity, marital status, BMI, sleep disorder, poverty income ratio, smoking status, alcohol consumption status, UIC.

### Subgroup analyses

Our study showed that sleep duration was negatively correlated with FT3 in thyroid function profiles, so we further evaluated the effect of sleep duration on FT3 in predefined and exploratory subgroups. Stratified analyses were performed as shown in Table 4. When sleep duration was used as a continuous variable, BMI and PIR had a significant interaction (p for interaction<0.05). With respect to BMI, overweight individuals (25 ≤ BMI < 30 kg/m2) had a greater decrease in FT3 levels compared to normal weight and obese individuals who slept longer. In terms of PIR, sleep duration was not associated with FT3 levels in low-income groups (PIR < 1), but there was a significant negative association in middle-income (1 ≤ PIR < 4) and high-income groups (PIR ≥ 4). When sleep duration was used as a categorical variable, PIR still had a significant interaction, and again sleep duration was significantly negatively associated with FT3 levels in middle-income and high-income populations (p for trend < 0.05), but not in low-income populations.

**Table 4:** Effect size of sleep duration on FT3 in prespecified and exploratory subgroups.

Character	Total sleep duration			Categories sleep duration				
	95% CI	*p*	*p* for interaction	<7	7–9	>9	*p* for trend	*p* for interaction
Sex			0.323					0.89
Male	-0.014(-0.028,0.001)	0.073		reference	-0.053(-0.095,-0.011)	-0.067(-0.159, 0.026)	0.01	
Female	-0.024(-0.038,-0.009)	0.002		reference	-0.058(-0.102,-0.013)	-0.102(-0.210, 0.006)	0.008	
AGE (years)			0.739					0.174
>50	-0.019(-0.030,-0.008)	0.001		reference	-0.038(-0.067,-0.008)	-0.146(-0.234,-0.059)	<0.001	
<50	-0.016(-0.030,-0.002)	0.029		reference	-0.059(-0.104,-0.014)	-0.057(-0.153, 0.039)	0.013	
Race/Ethnicity			0.087					0.243
White	-0.031(-0.048,-0.014)	<0.001		reference	-0.081(-0.128,-0.034)	-0.13(-0.238,-0.023)	<0.001	
Black	-0.012(-0.041,0.018)	0.435		reference	-0.015(-0.079, 0.050)	-0.239(-0.376,-0.103)	0.257	
Mexican	0.01(-0.027,0.048)	0.573		reference	-0.008(-0.175,0.159)	0.091(-0.196,0.379)	0.956	
Other	-0.012(-0.029,0.005)	0.164		reference	-0.04(-0.103,0.022)	-0.084(-0.183,0.015)	0.126	
Ever told doctor had trouble sleeping?			0.536					0.889
No	-0.024(-0.039,-0.008)	0.003		reference	-0.074(-0.119,-0.030)	-0.117(-0.204,-0.030)	<0.001	
Yes	-0.033(-0.057,-0.009)	0.008		reference	-0.067(-0.125,-0.010)	-0.14(-0.246,-0.033)	0.016	
Ever told by doctor have sleep disorder?			0.194					0.918
No	-0.019(-0.031,-0.006)	0.004		reference	-0.062(-0.099,-0.024)	-0.104(-0.180,-0.028)	<0.001	
Yes	-0.045(-0.083,-0.007)	0.020		reference	-0.078(-0.182,0.026)	-0.105(-0.340,0.129)	0.116	
Smoke			0.408					0.178
Never	-0.027(-0.043,-0.012)	<0.001		reference	-0.078(-0.134,-0.021)	-0.157(-0.248,-0.065)	0.003	
Former	-0.021(-0.039,-0.003)	0.026		reference	-0.026(-0.070, 0.018)	-0.187(-0.308,-0.065)	0.077	
Now	-0.012(-0.036,0.012)	0.318		reference	-0.054(-0.125,0.017)	0.013(-0.100,0.126)	0.187	
Alcohol. User			0.096					0.26
Never	-0.009(-0.027,0.009)	0.324		reference	-0.031(-0.106,0.044)	-0.071(-0.220,0.078)	0.3	
Former	-0.035(-0.062,-0.009)	0.010		reference	-0.088(-0.190, 0.013)	-0.143(-0.283,-0.004)	0.055	
Mild	-0.037(-0.052,-0.022)	<0.0001		reference	-0.093(-0.129,-0.057)	-0.157(-0.306,-0.008)	<0.0001	
Moderate	-0.012(-0.033,0.009)	0.252		reference	-0.024(-0.073,0.026)	-0.205(-0.469,0.058)	0.212	
Heavy	-0.003(-0.033,0.027)	0.842		reference	-0.001(-0.094,0.093)	-0.083(-0.280,0.113)	0.857	
BMI (kg/m2)			**0.013**					0.069
<25	-0.01(-0.030,0.010)	0.336		reference	-0.063(-0.126,0.001)	-0.067(-0.179,0.046)	0.042	
25–29.9	-0.041(-0.059,-0.023)	<0.0001		reference	-0.104(-0.164,-0.045)	-0.136(-0.266,-0.006)	<0.001	
≥30	-0.013(-0.029,0.003)	0.119		reference	-0.013(-0.068, 0.041)	-0.135(-0.260,-0.011)	0.369	
UIC (ug/dL)			0.285					0.189
<100	-0.019(-0.041,0.002)	0.078		reference	-0.046(-0.097,0.005)	-0.078(-0.203,0.048)	0.071	
100–299	-0.014(-0.028,-0.001)	0.032		reference	-0.05(-0.098,-0.003)	-0.073(-0.191, 0.045)	0.028	
≥300	-0.036(-0.060,-0.012)	0.004		reference	-0.122(-0.195,-0.048)	-0.141(-0.259,-0.022)	0.001	
PIR			**0.016**					**0.018**
<1	0.002(-0.015,0.020)	0.809		reference	0.014(-0.043,0.070)	0.025(-0.132,0.181)	0.598	
1–3.9	-0.025(-0.041,-0.009)	0.003		reference	-0.06(-0.109,-0.011)	-0.175(-0.265,-0.084)	0.003	
≥4	-0.035(-0.056,-0.014)	0.002		reference	-0.092(-0.146,-0.037)	-0.086(-0.234, 0.061)	<0.001	
Marital			0.068					0.171
Married, or living with partner	-0.026(-0.039,-0.013)	<0.001		reference	-0.074(-0.118,-0.031)	-0.055(-0.126, 0.016)	0.001	
Widowed, divorced, or separated	-0.039(-0.063,-0.015)	0.002		reference	-0.08(-0.142,-0.018)	-0.265(-0.373,-0.158)	0.001	
Never married	-0.004(-0.025,0.018)	0.725		reference	-0.032(-0.103,0.040)	-0.092(-0.233,0.050)	0.267	

Each stratification adjusted for all factors (sex, age, race/ethnicity, marital status, BMI, sleep disturbance, poverty income ratio, smoking status, alcohol consumption status, UIC) except the stratification factor itself.

## Discussion

This study was conducted with the goal of examining the relationship between sleep duration and thyroid function by analyzing data from a nationally representative cohort of adults in the US. These analyses revealed that sleep duration and FT3 levels were negatively correlated, with FT3 levels decreasing as sleep duration increased. When sleep duration was included as a categorical variable, FT3 levels were lower in the group with longer sleep duration. After adjusting for relevant confounders, this negative correlation remained, being primarily evident for participants who slept less than or equal to 7 hours, whereas for participants who slept more than 7 hours, FT3 levels did not change significantly with longer sleep duration. The present results also suggested an increase in TSH levels with increasing sleep duration, although this positive correlation was not significant after adjusting for confounders. No significant correlation was observed between other analyze thyroid parameters and sleep duration.

Multiple studies have investigated how sleep impacts thyroid function. A circadian variation in TSH secretion has been observed [20, 29]. Russell et al. also found similarly pronounced circadian rhythmicity for FT3, reporting that FT3 peaked 90 minutes following TSH in 86–100% of participants. In contrast, FT4 did not show a clear circadian rhythm [21], which may be because free T4 has a longer half-life (∼6.7 days). This also leads to a greater susceptibility of TSH and FT3 levels to sleep, which is also broadly similar to our study, where only TSH and FT3 levels were statistically different among sleep duration subgroups of analyzed thyroid function-related parameters, while FT4 was not. Recent studies have investigated the effects of sleep on TSH secretion based on relatively prolonged intervals of moderate sleep deprivation [25, 30]. These analyses repeatedly revealed that sleep deprivation contributed to the suppression of TSH secretion, although these analyses included relatively few subjects [23, 25]. Further studies confirming the link between sleep and thyroid function in a larger nationally representative cohort would thus be interesting.

Sleep and the endocrine system bidirectionally influence one another [31], and hormones and sleep are highly interdependent in the context of normal physiological function [32]. Indeed, sleep strongly influences thyroid hormone production, in turn impacting sleep duration and quality [16, 33]. The interaction between the HPT axis and the sleep cycle shapes metabolic processes and energy levels, and the disruption of one can lead to dysregulation of the other. Hyperthyroidism is a common cause of sleep disorders, and Stern et al. [34] conducted an analysis of 137 patients with Graves’ disease, which is the leading cause of hyperthyroidism, ultimately finding that difficulties falling asleep were reported by 66.4% of these individuals. Prolonged sleep latency was associated with TH-mediated changes in appetite, mood (i.e. increased anxiety), and bowel movements, ultimately contributing to reduced sleep duration. Xia et al. [35] found that TSH, T3, and T4 levels were directly correlated with insomnia symptom severity. Hyperthyroidism can also contribute to or aggravate anxiety, depression, and other conditions, thereby further impacting insomnia and sleep quality [36]. Hypothyroidism can similarly affect overall sleep quality, with multiple reports having documented a relationship between untreated subclinical hypothyroidism and poorer sleep. Song et al. [37] found that individuals exhibiting lower TH levels generally exhibited prolonged sleep latency, reduced sleep direction, and lower sleep quality as compared to people with normal thyroid function.

The study has several limitations. Firstly, as this was a cross-sectional analysis causal relationship could not be established with respect to the association between sleep and thyroid function. Future longitudinal research will be necessary to expand on these findings. Secondly, blood sampling for thyroid function was performed at individual time points during the morning period, and the blood was completed and processed and then cryopreserved before being sent together once a week to a designated laboratory for testing; therefore, differences in sampling time and storage time may affect thyroid function levels [38], and it may limit the interpretation of these results. In addition, participants’ sleep duration was based on self-reported durations rather than derived from a specific sleep quality scale, so the numbers provided may not accurately reflect their actual sleep duration, and these results may be affected by seasonal variation and recall bias [39]. Lastly, there may be additional confounding factors that were not appropriately accounted for. Even when taking these limitations, this is a population-based study of the association between sleep duration and thyroid function in US adults.

## Conclusion

This analysis revealed that when analyzing a nationally representative group of adults in the US, FT3 levels were significantly and negatively correlated with sleep duration when sleep duration was ≤7 hours. In contrast, when sleep duration exceeded 7 hours, FT3 levels did not change significantly with further increases in sleep duration.
